# Antifungal Activity and Major Bioactive Compounds of Water Extract of *Pangium edule* Seed against *Aspergillus flavus*

**DOI:** 10.1155/2021/3028067

**Published:** 2021-10-04

**Authors:** Kharisma I. Listyorini, Harsi D. Kusumaningrum, Hanifah N. Lioe

**Affiliations:** ^1^Food Science Study Program, Graduate School, IPB University, Bogor, Indonesia; ^2^Department of Food Science and Technology, Faculty of Agricultural Engineering and Technology, IPB University, Bogor, Indonesia

## Abstract

*Pangium edule* seeds are widely used as spices in Southeast Asia in a fresh and fermented form and are reported to have active compounds for food preservation. However, scientific data on the active compounds of *P. edule* seed that can prevent the growth of toxigenic *Aspergillus flavus* have not been widely reported. This research subjected to determine the antifungal activity and identify the active compounds of water extract of old and fermented seed of *P. edule* against *A. flavus*. The water extract was compared to the extracts obtained by multilevel maceration using 50% ethanol, ethyl acetate, and n-hexane as solvents. Alkaloid, saponin, phenolic compound, flavonoid, triterpenoid, and glycoside were detected qualitatively in the crude extracts. The water extract showed the best activity to suppress the growth of *A. flavus*, determined by the agar dilution method, with the minimum inhibitory concentration (MIC) of 12.5 and 25 mg/mL for old and fermented seed, respectively. The water extracts showed a moderate toxicity with LC_50_ of 100-500 *μ*g/mL, determined by the brine-shrimp toxicity test. After fractionation using 3 kDa molecular-weight (MW) cut-off ultrafiltration membrane, two fractions, i.e., fraction with MW < 3 kDa and >3 kDa, were obtained. The fraction with MW < 3 kDa showed the best antifungal activity with the MIC of 6.25 and 12.5 mg/mL for old and fermented seed, respectively. LC-MS/MS profile showed that different compounds belong to fatty acid, amino acid, glycoside, and peptide were found as major active compounds in the fractionated water extract. The principal compounds and partial least-square analysis, however, suggested that fatty acid and glycoside are responsible for the antifungal activity. Hence, this study concluded that the water extract of *P. edule* seed had promising antifungal activity against A. *flavus* which was due to presence of particular compounds belong to fatty acid and glycoside.

## 1. Introduction

*Pangium edule* is a traditional medicinal plant which its fruit-seed is often used as spices in Southeast Asian countries, including Indonesia. The fresh, old, and fermented seeds are widely used in different culinary recipes. The fermentation process of *P. edule* seed usually done by buried the boiled *P. edule* seeds in the ground for 40 days covered with husk ashes [1]. After fermentation, the seeds turn to a dark-brown product with slightly mushy texture.

Some studies have shown that *P. edule* seeds contain glycoside, unsaturated fatty acids, tannins, phenolic compounds, and alkaloid which have an inhibitory effect on the microbial growth [2–5]. Traditionally, the Indonesian people use *P. edule* seed to prolong the shelf-life of fishery products and using it as a fermentation medium [6]. Fresh and fermented *P. edule* seeds that have also been reported can be used as potential preservative in fresh fish by inhibiting the growth of pathogenic and histamine-producing microorganisms in fish [2, 7, 8]. The preservation of fishery products with *P. edule* seed suggested that *P. edule* seed has potential antifungal activity.

Previous studies have been conducted to assess the antifungal activity of *P. edule* seed against some fungi [9, 10]. Study using fresh seed showed that the water extract of fresh *P. edule* seed with concentrations of 20, 40, 60, 80, and 100% (*v*/*v*) could inhibit the growth of *Rhizoctonia* sp. The optimum inhibition was showed by 100% (*v*/*v*) water extract, with inhibition of 39.34%. This study also indicated that 30% (*v*/*v*) water extract of fresh *P. edule* seed could inhibit the growth of *Cylindrocladium* sp. [9]. Furthermore, another study reported that methanolic-extract of fresh *P. edule* seed with a concentration of 15% could optimally inhibit the growth of *Aspergillus flavus* [10]. However, the data on the antifungal activity and the identified active compounds of water extract of *P. edule* against *A. flavus* are still limited. *A. flavus* has been known as aflatoxin producer, a toxin that can affect the mechanism of the liver in humans, mammals, and poultry, thus becoming a factor causing liver cancer [11, 12]. *A. flavus* that has been reported is also found in the preserved fishery products, such as semidried and salted fish products [13, 14]. Hence, this research is aimed at determining the antifungal activity of water extract of old and fermented seed of *P. edule* against toxigenic *A. flavus*. The fractionated extracts by ultrafiltration membrane were also studied, and the major active compounds were identified by LC-MS/MS.

## 2. Materials and Methods

### 2.1. Sample Collection and Preparation

Old (dark-brown color) *P. edule* seeds were collected from Padang Pariaman, Padang, West Sumatra. Fermented *P. edule* seeds were collected from Nganjuk, East Java. Both samples were collected in 2019. The collected seeds were peeled manually and air-dried for 2 days. The dried seeds were then crushed to obtain powdered *P. edule* seed. The powdered sample were kept in plastic bags and transported to the laboratory for analysis.

### 2.2. Physico-Chemical Property Assay

Proximate analysis was carried out using the standard method, including moisture content (AOAC, 2012; method 935.29), ash content (AOAC, 2012; method 942.05), protein content (AOAC, 2012; method 960.52) [15], fat content (AOAC, 2005; method 2003.06) [16], and carbohydrate content (by difference). Color analysis was performed using a Chromameter (Konica Minolta, CR-300).

### 2.3. Sample Extraction

The extraction process was carried out by multilevel maceration methods using water, 50% ethanol, ethyl acetate, and n-hexane as solvents. Seventy-five grams of both *P. edule* seed powders, i.e., old and fermented seed, was macerated using orbital shaker (New Brunswick, Germany) for 72 hours at a weight ratio of the sample, and the volume of solvent was 1 : 4. The extracts obtained from maceration processes were filtered using Whatman grade 1 filter paper and concentrated using vacuum rotary evaporator (Buchi, Switzerland) at high pressure at 50-60°C. The extracts that have been obtained then be stored in a freezer with a temperature of -15 to -20°C until it was used for analysis.

### 2.4. Qualitative Phytochemical Screening

The *P. edule* crude seed extracts were used to perform the phytochemical analysis. The various natural compounds present in the extracts were determined qualitatively using the methods for the detection of alkaloid, saponin, tannin, phenolic, flavonoid, triterpenoid, steroid, and glycoside [17, 18].

### 2.5. Determination of Antifungal Activity

Pure culture of *Aspergillus flavus* was maintained on slant of potato dextrose agar (PDA, Oxoid, UK) at 4°C. Prior to the testing, *A. flavus* was subcultured by transferring a loop of cells onto slant of PDA medium and incubated for five days at 28°C, and the spores were harvested in sterilized saline solution (NaCl 0.85%) [19]. The crude extracts that have been obtained was dissolved in 100% dimethyl sulfoxide (DMSO, Merck, Germany) and prepared as stock solutions, and serial twofold dilutions were performed. The final concentrations of the extracts ranged from 8 to 250 mg/mL. The antifungal activity of the extracts against *A. flavus* was measured using the agar dilution method [20, 21]. These extracts were diluted 1 : 10 in plates containing melted PDA medium to obtain the test concentrations from 0.8 to 25 mg/mL and a final DMSO concentration 10%. The harvested spores of *A. flavus* were then inoculated into these plates, after the medium had solidified, in 32 spots. The plates were incubated at 28°C for three days. Dimethyl sulfoxide (DMSO) 100% was tested as a negative control. The MIC value was read as the lowest concentration showing 100% growth inhibition. Each assay was conducted in three replicates.

### 2.6. Fractionation of Water Extract of P. edule Seed

Fractionation was performed according to Andayani et al. [22], with slight modification. The water extract was dissolved in DMSO to obtain an extract concentration of 500 mg/mL. The extract solution was then centrifuged (Hermle Z383-K, Germany) at 1600 × g for 30 minutes at room temperature and filtered using a glass microfiber filter (Whatman GF/A Glass Circles, 1.6 *μ*m pore size, 110 mm, UK). Then, the extract solution was filtered again using nylon membrane filter (nylon 25 mm; 0.22 *μ*m pore size, Aijiren, China). Each of 20 mL of extract solution obtained was fractionated using the 3 kDa MWCO (molecular weight cut off) ultrafiltration membrane (Amicon Ultra-15 3 K Centrifugal Filter Unit, Ultracel-3 regenerated cellulose membrane, Merck Millipore, USA) for 30 minutes at 3900 × g, 28°C. Two fractions were obtained, the fraction with molecular weight (MW) of <3 kDa and >3 kDa. Each fraction was adjusted to the final volume of 20 mL using DMSO. Antifungal activity was tested for the both fractions by agar dilution method. The fraction which had the best inhibitory activity was defined as the fraction that showed the lowest MIC [20, 21].

### 2.7. LC-MS/MS Analysis

The fraction which showed the best inhibitory activity was analyzed using LC-MS/MS. The samples (10 mg) were dissolved in 5 mL methanol and filtered using PTFE membrane filter (PTFE 25 mm; 0.22 *μ*m; pore size, Anpel, China). An amount of 2 *μ*L sample was injected into LC-MS/MS with a flow rate of 0.2 mL/min. LC-MS/MS analysis was performed on a Thermo Scientific Vanquish Flex Binary UHPLC (Thermo Fisher Scientific, USA) connected to a Thermo Scientific Q Exactive Plus Orbitrap High Resolution Mass Spectrometer (Thermo Fisher Scientific, USA). The mass spectrometer was equipped with electrospray ionization (ESI) in the positive mode. The MS/MS spectra were obtained with a mass range of *m/z* 100-1500. The chromatographic separation of the sample was performed on a C18 column (100 mm × 2.1 mm; 1.5 *μ*m, Accucore C18, Thermo Fisher Scientific, USA). The mobile phase consisted of water containing 0.1% formic acid for solvent A and acetonitrile containing 0.1% formic acid for solvent B. The gradient elution was 0-1 min (5% B), 1-25 min (5-95% B), 25-28 min (95% B), and 28-30 min (5% B).

### 2.8. Brine Shrimp Lethality Assay

*Artemia salina* Leach eggs were hatched in artificial sea water by dissolving 27 g commercial sea-salt in 3 L of water. After 48 hours, the shrimps matured as nauplii and were ready for the assay. The brine shrimp lethality test was carried out on the water extract of *P. edule* seed using the standard procedure [23, 24]. Ten milligrams of the extract was dissolved in 1 mL of water to give an extract concentration of 10 mg/mL as stock solution. Concentrations of 1 mg/mL, 500 *μ*g/mL, 100 *μ*g/mL, and 10 *μ*g/mL were prepared by making a serial dilution from the stock solution. A suspension of nauplii, containing 10 nauplii, was added into each test tube, and the number of dead nauplii was counted after 24 hours. Each assay was conducted in three replicates. Lethal concentration (LC_50_) was determined using the probit analysis method by SPSS 24.

### 2.9. Statistical Analysis

Data generated from the antifungal activity analysis were processed with SPSS 24 to perform one-way analysis of variance (ANOVA) using Tukey's multiple comparisons. Differences were considered statistically significant when *p* < 0.05. The data were expressed by means ± standard deviation. The categorization and contribution of compounds identified by LC-MS/MS analysis were analyzed by principal component analysis (PCA) and partial least-square regression (PLSR) using XLSTAT.

## 3. Results and Discussion

### 3.1. Proximate and Color of Seed Powders and the Yield of Extraction

The fermented *P. edule* seed showed higher moisture and protein content but lower in carbohydrate content (*p* < 0.05) than the old *P. edule* seed (Table 1). The differences were most likely attributed to the fermentation process, where the breakdown of chemical components occurs naturally. Microorganisms that are involved in the spontaneous fermentation process of *P. edule* seed thought to have amylolytic properties, indicated by the decrease in carbohydrate content in the fermented seeds. A previous study reported that fresh *P. edule* seed contains about 52% water (wet basis), 16% fat, 18% carbohydrate, and 13% protein [8].

The L∗ value ranges between 0 and 100, with 0 pointing black/dark color and 100 pointing to white/light. The more positive the a∗ value, the sample tends to be red, while the more negative the a∗ value, the sample tends to be green. The more positive the b∗ value, then the sample tends to be yellow, while the more negative the b∗ value, the sample tends to be blue. As shown in Table 1, the color of *P. edule* seed powders had a low brightness intensity and was a mixture of red and blue since it had positive a∗ value and negative b∗ value. Brightness intensity in old *P. edule* seed (23.70) was significantly higher than that of fermented *P. edule* seed (18.92) (*p* < 0.05).

**Figure 1:**
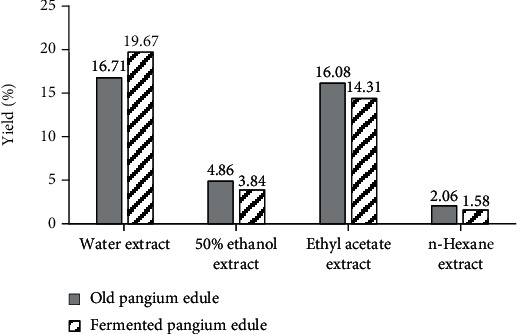
Yield of *Pangium edule* crude seed extracts.

The *P. edule* seeds were peeled manually and air-dried for 2 days before the extraction which yielded 24% dry material. The dried seeds were then crushed to obtain powdered *P. edule* seed, and 75 g of both powdered seeds was extracted using the maceration method. The multilevel extraction process using the maceration method was chosen because this method can produce large amounts of extract and avoid chemical changes due to heating [25]. The extraction of old and fermented *P. edule* seed with water solvent resulted in the highest yield extract value, i.e., 16.71% and 19.67%, respectively (Figure 1). However, the yield of ethyl acetate extract was relatively high due to the oil in *P. edule* seed was also extracted. Proximate analysis showed that the fat or oil content of old and fermented *P. edule* seed was relatively high, i.e., 42.38% and 41.83%, respectively. The oil in *P. edule* seed could be extracted when the seeds were macerated using ethyl acetate solvent since oil can dissolve in ethyl acetate. It causes the ethyl acetate extract not be able to fully dried by evaporative process.

Heruwati et al. [8] reported that the extract-yield of fresh *P. edule* seed using water, 50% ethanol, and n-hexane was 2.46%, 2.72%, and 0.54%, respectively. On the other hand, extraction of fermented *P. edule* seed using water, 50% ethanol, and n-hexane gave the yield of 7.72, 10.26, and 0.56%, respectively. It shows that the results of both studies showed same trend, i.e., the polar solvent gave high yield.

### 3.2. Qualitative Phytochemicals of Seed Extracts

Alkaloid was present in water and ethanolic seed extracts, while tannin and steroid were absent in all *P. edule* crude seed extracts (Table 2). All extracts contain saponin, phenolic compounds, flavonoid, and triterpenoid as well as glycosides. Phytochemicals are nonnutritive chemical compounds in plants that have protective or preventive properties against disease which are beneficial to human health [26].

These results have some similarities with previous study. Alkaloid was also found in crude extract of fermented *P. edule* seeds using water, 70% ethanolic, acetone, and n-hexane as solvents, while saponin, flavonoid, and tannin were found in water and ethanolic extracts [27]. Triterpenoid and steroid were not found in all *P. edule* seed crude extracts. Furthermore, phenolic compounds and condensed-tannin in mature *P. edule* seed were found in water and ethanolic extracts as reported by Makagansa et al. [5]. The differences may be caused by the origin of *P. edule* seeds and the extraction process [28].

### 3.3. Antifungal Activity of Extracts and Fractionated Extract

**Figure 2:**
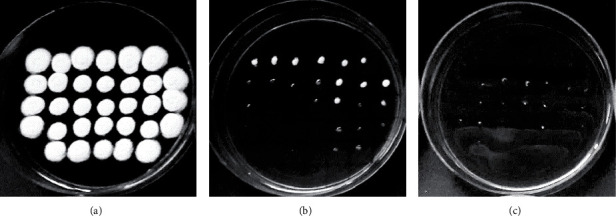
Growth of *A. flavus* on plates exposed to crude water extract of fermented *P. edule* seed, after three days incubation at 28°C: (a) negative control (100% DMSO); (b) 3.12 mg/mL; (c) 25 mg/mL.

Detectable growth of *A. flavus* by agar-dilution method was observed after three days incubation on plate without extract (control), while with 25 mg/mL water extract of fermented *P. edule* seed the growth was apparently inhibited (Figure 2). As shown in Figure 2, higher concentration of the *P. edule* crude seed extracts resulted in fewer and smaller visible mold spots on the plate, indicating that fewer fungal spores were able to germinate and 100% DMSO used as negative control showed no activity, indicated by visible growth of all mold spots (32 mold spots). Many studies have been used the agar dilution method to assess the antifungal activity of plant extracts. The main advantage of this method is provision of uniform and stable dispersion of extracts when they are incorporated into the agar medium. Another advantages of agar dilution method include the allowing of the qualitative and quantitative determinations to be carried out together; accurate determination of minimum inhibitory concentrations (MICs); allowing the determination of the antimicrobial resistance level, and potential to extend the antimicrobial concentration as far as required [29, 30].

The result showed that the concentration of the *P. edule* crude seed extracts and the type of solvent affected the growth inhibition of *A. flavus* (Table 3). The growth of *A. flavus* was reduced with the increase of crude extract concentration, indicated by a decrease on the number of visible mold spots growing on the plate. This reduction, however, only occurred on the plates with water and 50% ethanolic extracts. No inhibition was observed on the plates with ethyl acetate and n-hexane extracts. The water extract of old and fermented seed was found as the two-best inhibitor to the growth of *A. flavus* (*p* < 0.05).

Furthermore, the fractions that were obtained from water extract of old and fermented seed, i.e., the fraction with MW of <3 kDa and MW of >3 kDa, also showed antifungal activity against *A. flavus* (Table 3). The fraction with MW < 3 kDa showed a stronger antifungal activity than the fraction with MW > 3 kDa. This fraction also exhibited a relatively stronger antifungal activity compared to the unfractionated one, indicated by a lower concentration that was needed in achieving 100% inhibition.

### 3.4. Minimum Inhibitory Concentration (MIC)

The MIC, described as the lowest concentration showing 100% growth inhibition, was only determined for water extract and its fraction with MW < 3 kDa (Table 4). As shown in Table 3, apparent mold spots were still found on all plates of the other extracts in a concentration between 0.8 mg/mL and 25 mg/mL. The MIC_50_ and MIC_90_, that indicated, respectively, ≥50% and ≥90% inhibition, were also determined to facilitate the comparison between antifungal activity levels of *P. edule* seed extracts [20, 31].

As described in Table 4, the MIC shown by the water extract of old seed was lower than that of fermented seed. The fraction with MW of <3 kDa of old seed, however, showed the best inhibitory activity than the other samples (*p* < 0.05), indicated by the lowest MIC. The results are in line with the previous studies which indicated that *P. edule* seed extracted using polar solvents could inhibit the growth of *A. flavus* [10]. Some studies have shown that low MW compounds have a contribution to antifungal activity [32–35].

### 3.5. Identified Compounds in Fractionated Extract

**Figure 3:**
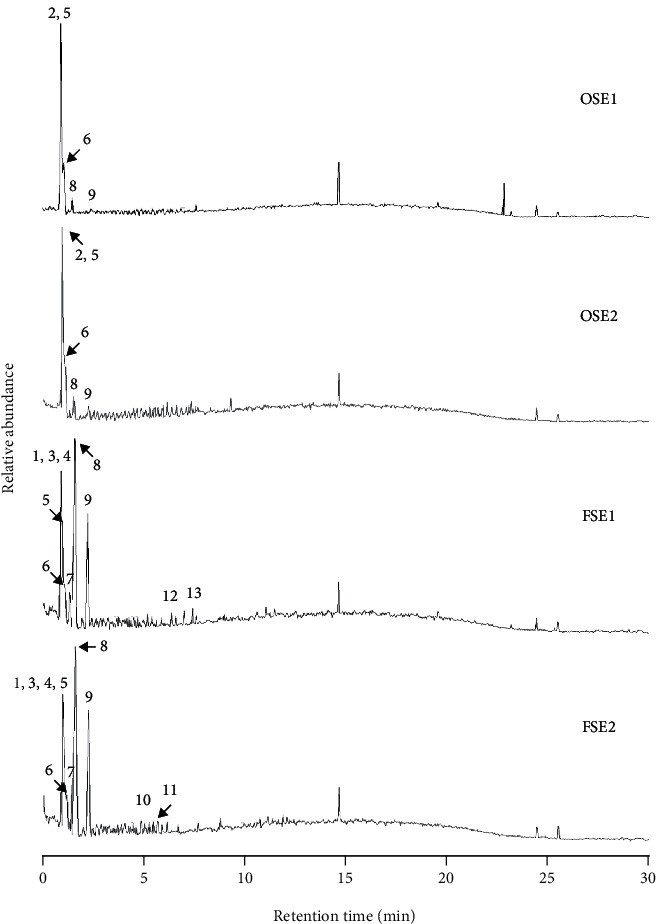
LC-MS/MS chromatogram of fraction < 3 kDa of water extract of old (OSE) and fermented (FSE) of *P. edule* seed (positive ion mode).

**Figure 4:**
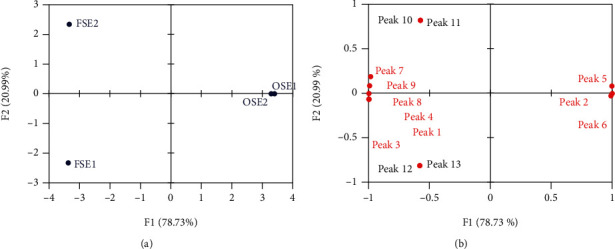
PCA results of the fraction < 3 kDa of water extract of old and fermented *P. edule* seeds: (a) score plot; (b) loading plot.

The antifungal compound profiles of fraction < 3 kDa of old and fermented seeds which found as the two-best inhibitor for *A. flavus* were analyzed using LC-MS/MS instrument (Figure 3). Thirteen major compounds were detected on the chromatogram and identified based on the retention time and MS data (molecular mass, *m/z* value of the MS/MS fragments) (Table 5). Based on the results of this analysis, the chemical profiles of fraction < 3 kDa of water extract of old and fermented *P. edule* seeds were identified as fatty acids (peaks 2, 3, and 6), amino acids (peaks 1, 4, 7, 8, and 9), glycoside (peak 5), and peptides (peaks 10-13). Furthermore, the description of the fraction < 3 kDa of water extract of old and fermented *P. edule* seeds towards the chromatogram peaks of the LC-MS/MS and its antifungal activity was visualized using principal component analysis (PCA) (Figure 4). F1 and F2 represented 99.72% of total data variance, with F1 covered for 78.73% of the data.

The PCA score plot discriminated OSE1 and OSE2 from other samples by separate cluster on the positive score value of F1, which showed the similar characteristics as antifungal activity (Figure 4(a)). The loading plot (Figure 4(b)) of PCA result showed that the most discriminatory constituents in OSE1 and OSE2 were peaks 2, 5, and 6 which presented in high amounts in OSE1 and OSE2 compared to in FSE1 and FSE2. Loading plot also showed that peaks 12 and 13 suggested as discriminant compounds in FSE1, while FSE2 formed a separate cluster with peaks 10 and 11 as discriminant compounds.

Contribution and correlation of each peak to the antifungal activity were described by variable importance in projection (VIP) scores and correlation coefficient (*r*) using partial least squares regression (PLSR) analysis. The result of PSLR indicated that peaks 2, 5, and 6 were contributed to the antifungal activity of water extract of old *P. edule* seed (VIP score > 1) and showed a positive correlation to antifungal activity (*r* > 0.90) (Figure 4(b)). Meanwhile, peaks 1, 3, 4, 7, 8, and 9 were contributed to the antifungal activity of water extract of fermented *P. edule* seed (VIP score > 1) but showed a negative correlation to antifungal activity (*r* < 0). Moreover, the result of PSLR also indicated that peaks 10, 11, 12, and 13 were not contributed to the antifungal activity of water extract of old and fermented *P. edule* seed. The different compounds present in the different extracts cannot be described by the result as shown in Table 2.

Based on the LC-MS/MS chromatogram in Figure 3, from the three peaks that contributed and are positively correlated on antifungal activity (peaks 2, 5, and 6), there were two peaks that were detected and identified in both water extract of old *P. edule* seed (OSE) and water extract of fermented *P. edule* seed (FSE), i.e., peaks 5 and 6 which were classified as glycoside and fatty acid. Peak 2 which classified as fatty acid was only detected and identified in water extract of old *P. edule* seed. This indicated that the peaks (compounds) responsible for the antifungal activity of the water extract of old *P. edule* seed are peaks 2, 5, and 6, while the peaks (compounds) responsible for the antifungal activity of the water extract of fermented *P. edule* seed are only peaks 5 and 6.

Overall, the PCA and PSLR analysis revealed that peptides were not contributed to the antifungal activity of the fraction < 3 kDa of water extract of old and fermented seeds. All of contributed peaks in this study classified as fatty acid, amino acid, and glycoside compounds (Table 5). Amino acids showed a negative correlation to the antifungal activity of the fraction < 3 kDa of water extract of old and fermented seeds, while fatty acids and glycoside showed a positive correlation to its antifungal activity. Therefore, both fatty acids and glycoside were compounds that are responsible for the antifungal activity of the fraction < 3 kDa of water extract of old and fermented *P. edule* seeds. Previous studies have been reported that fatty acids showed antifungal activity against pathogenic fungi, such as *Candida albicans*, *Aspergillus flavus*, and *Rhizopus nigricans* [36, 37]. Fatty acids induce fungal cell inhibition through cell membrane which is a major target for these compounds. Fatty acids can increase the membrane fluidity and cause a generalized disorganization of the cell membrane. It leads to conformational changes in membrane proteins which will result in leakage of the intracellular components and cell death [38, 39].

Moreover, the PCA and PSLR results also showed that glycosides contributed to antifungal activity of water extract of old and fermented *P. edule* seed. Glycosides have been reported to have antifungal activity against *T. mentagrophytes*, *C. albicans*, *B. cinerea*, *A. alternata*, *A. flavus*, *Aspergillus niger*, *Aspergillus ochraseus*, *Aspergillus versicolor*, *Penicillium funiculosum*, *Rhizoctonia cerealis*, and *Trichoderma viride* [40–43]. Zhang et al. [44] revealed that mechanism of antifungal properties of glycosides was damaging the plasma membrane and cause the leakage of cytoplasmic materials which leads to cell death.

### 3.6. The Median Lethal Concentration (LC_50_)

The LC_50_ determined by the brine-shrimp lethality test (BSLT) for water extract of *P. edule* seed is given in Table 6. The result shows that the water extracts are moderately toxic to *A. salina* (LC_50_: 468 *μ*g/mL for old seed and 244 *μ*g/mL for fermented seed). The toxicity based on the BSLT results is classified as follows: LC_50_ 0-100 *μ*g/mL, highly toxic; LC_50_ 100-500 *μ*g/mL, moderately toxic; LC_50_ 500-1000 *μ*g/mL, low toxic; and LC_50_ > 1000 *μ*g/mL, nontoxic [45]. According to Meyer's research, the plant extracts with LC_50_ of 30-1000 *μ*g/mL had potential as antimicrobial agent and pesticide [23].

The toxicity test of the seed water extracts against *A. salina* larvae has not been reported before, though Sudjana et al. [46] and Simanjuntak et al. [47] have described the toxicity of *P. edule* seed extract with other solvents. The methanolic and chloroform extracts of *P. edule* seed had LC_50_ of 274.26 *μ*g/mL and 916.13 *μ*g/mL, respectively, while the n-hexane extract was nontoxic since it had LC_50_ > 1000 *μ*g/mL [46]. Furthermore, the ethanolic extract of fresh *P. edule* seed was also considered as nontoxic against *A. salina* larvae (LC_50_ > 1000 *μ*g/mL) as reported by Simanjuntak et al. [47]. The differences in the LC_50_ value can be due to different type of *P. edule* used which cause differences in the secondary metabolites produced. In addition, different extraction methods and solvents also affect the dissolved active compounds, causing differences in biological activities of same plant species [48, 49].

Previous studies reported that LC_50_ of the BSLT and the LD_50_ of the acute oral toxicity assay in animal models has a positive correlation [45, 50]. According to Parra's research, the brine shrimp LC_50_ < 10 *μ*g/mL has LD_50_ 100-1000 mg/kg; LC_50_ < 20 *μ*g/mL has LD_50_ 1000-2500 mg/kg; LC_50_ > 25 *μ*g/mL has LD_50_ 2500-8000 mg/kg [50]. We can assume that the LD_50_ of oral acute toxicity for water extract of old and fermented of *P. edule* seed also will be more than 2500 mg/kg, because the LC_50_ of BSLT is 468 and 244 *μ*g/mL, respectively. The chemical labeling and classification of acute systemic toxicity based on oral LD_50_ are recommended by the Organization for Economic Co-operation and Development (OECD) as follows: ≤ 5 mg/kg, very toxic; 5-50 mg/kg, toxic; 50-500 mg/kg, harmful; and 500-2000 mg/kg, no label [51]. Hence, this finding showed that the water extract of *P. edule* seed could be developed as an antifungal or preservative agent since the oral LD_50_ suggests that the extract is nontoxic.

## 4. Conclusions

This study found that the water extract fraction with MW < 3 kDa of old *P. edule* seed showed the best inhibitory activity against *A. flavus*, followed by the fraction with MW < 3 kDa of fermented seed. These fractions showed a stronger antifungal activity than the unfractionated water extract, indicated by the lower MIC. The responsible compounds for the antifungal activity of water extract of old and fermented seed were classified into the same group, i.e., fatty acid and glycoside. Purification and confirmation of the antifungal activity of the purified compound were included in the further study.

## Figures and Tables

**Table 1:** Proximate and color of *P. edule* seed powders (dry weight basis).

Parameter	Fermented seed	Old seed
Moisture (%)^a^	9.09 ± 0.72	7.41 ± 0.56
Ash (%)	3.56 ± 0.15	3.36 ± 0.29
Protein (%)^a^	24.96 ± 0.09	20.17 ± 0.15
Fat (%)	41.83 ± 0.42	42.38 ± 0.26
Carbohydrate (%)^a^	29.65 ± 0.56	34.08 ± 0.41
L^∗^^a^	18.92 ± 3.12	23.71 ± 1.23
a^∗^^a^	3.30 ± 0.28	5.87 ± 0.31
b^∗^	−5.49 ± 0.31	−5.49 ± 1.20

L^∗^ indicates the level of brightness; a^∗^ and b^∗^ values indicate the color trend. Super indexes of letter a mean significantly different between the extracts (*p* < 0.05).

**Table 2:** Qualitative phytochemical screening of *Pangium edule* crude seed extracts.

Compounds	Water		50% ethanol		Ethyl acetate		n-Hexane	
	OSE	FSE	OSE	FSE	OSE	FSE	OSE	FSE
Alkaloid	+	+	+	+	-	-	-	-
Saponin	+	+	+	+	+	+	+	+
Tannin	-	-	-	-	-	-	-	-
Phenolic	+	+	+	+	+	+	+	+
Flavonoid	+	+	+	+	+	+	+	+
Triterpenoid	+	+	+	+	+	+	+	+
Steroid	-	-	-	-	-	-	-	-
Glycoside	+	+	+	+	+	+	+	+

OSE: old *Pangium edule* seed extract; FSE: fermented *Pangium edule* seed extract; (+): present; (-): not present.

**Table 3:** Antifungal activity of extracts and fractionated water extract of *P. edule* seed against *A. flavus* as determined by agar dilution.

Seed	Extraction solvent	Number of detected mold spot at different concentrations						
		25 mg/mL	12.5 mg/mL	6.25 mg/mL	3.12 mg/mL	1.6 mg/mL	0.8 mg/mL	100% DMSO (control)
Old seed	Water	0 ± 0.0^a^	0 ± 0.0^a^	4 ± 3.2^a^	21 ± 5.5^c^	29 ± 2.7^ef^	31 ± 1.3^f^	32 ± 0.0^f^
	50% ethanol	3 ± 2.9^a^	4 ± 3.9^a^	13 ± 2.5^b^	19 ± 2.6^c^	22 ± 2.7^cd^	26 ± 4.5^de^	
	Ethyl acetate	32 ± 0.8^f^	32 ± 0.8^f^	31 ± 2.4^f^	32 ± 0.4^f^	32 ± 0.5^f^	32 ± 0.5^f^	
	n-Hexane	32 ± 0.0^f^	32 ± 0.8^f^	32 ± 0.0^f^	32 ± 0.0^f^	32 ± 0.0^f^	32 ± 0.4^f^	
Fermented seed	Water	0 ± 0.0^a^	3 ± 3.6^ab^	8 ± 2.0^bc^	16 ± 2.9^de^	23 ± 2.5^fgh^	30 ± 1.6^i^	32 ± 0.0^i^
	50% ethanol	3 ± 2.3^ab^	4 ± 4.0^ab^	11 ± 2.0^cd^	19 ± 4.9^ef^	21 ± 5.6^efg^	27 ± 4.4^ghi^	
	Ethyl acetate	29 ± 4.0^i^	29 ± 4.8^hi^	31 ± 1.7^i^	31 ± 2.1^i^	31 ± 1.5^i^	31 ± 1.1^i^	
	n-Hexane	32 ± 0.0^i^	32 ± 0.8^i^	32 ± 0.0^i^	32 ± 0.4^i^	32 ± 0.0^i^	32 ± 0.0^i^	
Old seed	Water, <3 kDa	0 ± 0.0^a^	0 ± 0.0^a^	0 ± 0.0^a^	14 ± 3.2^b^	18 ± 1.8^bcd^	18 ± 1.6^bcd^	32 ± 0.0^e^
	Water, >3 kDa	14 ± 3.0^b^	15 ± 3.1^bc^	16 ± 4.7^bc^	17 ± 4.1^bcd^	19 ± 3.2^cd^	21 ± 2.0^d^	
Fermented seed	Water, <3 kDa	0 ± 0.4^a^	0 ± 0.0^a^	15 ± 2.8^b^	17 ± 2.8^bcde^	18 ± 2.1^bcde^	20 ± 2.6^de^	32 ± 0.0^f^
	Water, >3 kDa	15 ± 1.6^b^	16 ± 1.6^bc^	17 ± 1.9^bcd^	18 ± 1.2^bcde^	19 ± 2.5^cde^	21 ± 2.8^e^	

Numbers are means of three replications. Numbers followed by different letters in each row of *P. edule* seed show a significant difference (*p* < 0.05) within different extract concentration and also between control.

**Table 4:** The MIC, MIC_90_, and MIC_50_ that were determined using the agar dilution method.

Seed	Sample	MIC	MIC_90_	MIC_50_
Old seed	Water extract	12.5 mg/mL	12.5 mg/mL	6.25 mg/mL
	50% ethanolic extract	—	25 mg/mL	6.25 mg/mL
	Water extract fraction < 3 kDa	6.25 mg/mL	6.25 mg/mL	3.12 mg/mL
Fermented seed	Water extract	25 mg/mL	12.5 mg/mL	3.12 mg/mL
	50% ethanolic extract	—	25 mg/mL	6.25 mg/mL
	Water extract fraction < 3 kDa	12.5 mg/mL	12.5 mg/mL	6.25 mg/mL

MIC: minimum inhibitory concentration, the lowest concentration showing 100% growth inhibition; MIC_50_ and MIC_90_ indicated ≥50% and ≥90% inhibition, respectively.

**Table 5:** Compounds identified in the water extract of old and fermented *Pangium edule* seed by LC-MS/MS analysis.

Peak no.	Identified compounds	Rt (mins)	Precursor ion (*m/z*)	Product ions (*m/z*)	Formula [M-H]^+^	MW (g/mol)	Detected samples
1	1-[(3-Carboxypropyl)amino]-1-deoxy-beta-D-fructofuranose	0.99	266.1228	248	C_10_H_19_NO_7_	265.1156	FSE1, FSE2
2	Methyl (4Z)-5-(1,3-dioxolan-2-yl)-2-hydroxy-4-(hydroxyimino)pentanoate	1.00	234.0970	216 188 102 97 90	C_9_H_15_NO_6_	233.0900	OSE1, OSE2
3	Ethylenediamine-N,N′-diacetic-N,N′-dipropionic acid	1.02	321.1285	160 124 84	C_12_H_20_N_2_O_8_	320.1213	FSE1, FSE2
4	2′-Deoxymugineic acid	1.06	305.1332	130 84	C_12_H_20_N_2_O_7_	304.1260	FSE1, FSE2
5	Methyl 4,6-dideoxy-4-[(2,4-dihydroxybutanoyl)amino]-2-O-methylhexopyranoside	1.09	294.1543	276 230 88	C_12_H_23_NO_7_	293.1465	OSE1, OSE2, FSE1, FSE2
6	Methyl (5-acetamido-2,2-dimethyl-4,6-dioxo-1,3-dioxan-5-yl)acetate	1.22	274.0916	256 238 130 97 84	C_11_H_15_NO_7_	273.0843	OSE1, OSE2, FSE1, FSE2
7	6-Hydroxypicolinic acid	1.40	140.0340	112	C_6_H_5_NO_3_	139.0267	FSE1, FSE2
8	Isoleucine	1.56	132.1017	86	C_6_H_13_NO_2_	131.0944	OSE1, OSE2, FSE1, FSE2
9	Phenylalanine	2.27	166.0860	120	C_9_H_11_NO_2_	165.0787	OSE1, OSE2, FSE1, FSE2
10	Leucyl-valine	4.83	231.1696	72	C_11_H_22_N_2_O_3_	230.1624	FSE2
11	Leucyl-alanyl-proline	5.67	300.1909	116 86	C_14_H_25_N_3_O_4_	299.1836	FSE2
12	Leucyl-aspartyl-valine	6.40	346.1963	215 187 72	C_15_H_27_N_3_O_6_	345.1890	FSE1
13	Phenylalanyl-valyl-aspartic acid	7.37	380.1805	215 187 120 72	C_18_H_25_N_3_O_6_	379.1732	FSE1

**Table 6:** LC_50_ by the brine-shrimp lethality test of water extract of *P. edule* seed.

Extracts	Concentration (*μ*g/mL)	Mortality (%)	LC_50_ (*μ*g/mL)
Old seed extract	1000	80.00	468
	500	53.33	
	100	26.67	
	10	23.33	
Fermented seed extract	1000	90.00	244
	500	60.00	
	100	43.33	
	10	36.67	

## Data Availability

All datasets used to support the findings of this study are available upon reasonable request from the corresponding author.
